# ASSESSING RISK FOR ENDOMETRIAL CANCER AMONG HISPANIC FEMALES AGE 50 YEARS AND OLDER

**DOI:** 10.1093/geroni/igz038.1720

**Published:** 2019-11-08

**Authors:** Ana Rodriguez, Yong-Fang Kuo, Enshuo Hsu

**Affiliations:** 1 The University of Texas Medical Branch at Galveston, Galveston, Texas, United States; 2 University of Texas Medical Branch, Galveston, Texas, United States; 3 UTMB, Gaveston, Texas, United States

## Abstract

Endometrial cancer is the most common gynecological cancer in the US, with most women diagnosed between 55 and 64 years old. Seventy-five percent of women with endometrial cancer are postmenopausal, and the most common symptom is postmenopausal bleeding. Only a few studies have addressed the lack of knowledge and awareness of risk factors and/or health care utilization for early signs and symptoms of endometrial cancer. The objective of this study was to evaluate health care utilization among Hispanic women aged ≥50 years who are at risk for endometrial cancer. This retrospective cohort study used a combination of diagnosis and procedure codes from UTMB’s electronic health records to identify Texas Hispanic females who had a health encounter at ≥50 years of age between 2012 and 2016. Risk factors included conditions/treatments affecting hormone levels, age, body mass index, diabetes, gravidity, parity, family history of endometrial or colorectal cancer, previous diagnosis of breast or ovarian cancer or endometrial hyperplasia, smoking or alcohol use, and treatment with radiation therapy in the pelvis area. Multivariate logistic regression models evaluated for predictors of endometrial cancer. The study included 11,563 Hispanic females aged ≥50 years (median age=57). Most women were overweight. Currently, we identified 705 Hispanic females (6.1%) with possible endometrial cancer with validation underway. Females who have a history of vaginal spotting/bleeding, pelvic bleeding, and pelvic pain are at higher risk for endometrial cancer. It is important for physicians to educate patients on recognizing the signs and symptoms of endometrial cancer.

